# In search of causal variants: refining disease association signals using cross-population contrasts

**DOI:** 10.1186/1471-2156-9-58

**Published:** 2008-08-29

**Authors:** Nancy L Saccone, Scott F Saccone, Alison M Goate, Richard A Grucza, Anthony L Hinrichs, John P Rice, Laura J Bierut

**Affiliations:** 1 Department of Genetics, Washington University, Campus Box 8232, 4566 Scott Avenue, Saint Louis, Missouri, USA; 2 Department of Psychiatry, Washington University, Box 8134, 660 South Euclid Avenue, Saint Louis, Missouri, USA

## Abstract

**Background:**

Genome-wide association (GWA) using large numbers of single nucleotide polymorphisms (SNPs) is now a powerful, state-of-the-art approach to mapping human disease genes. When a GWA study detects association between a SNP and the disease, this signal usually represents association with a set of several highly correlated SNPs in strong linkage disequilibrium. The challenge we address is to distinguish among these correlated loci to highlight potential functional variants and prioritize them for follow-up.

**Results:**

We implemented a systematic method for testing association across diverse population samples having differing histories and LD patterns, using a logistic regression framework. The hypothesis is that important underlying biological mechanisms are shared across human populations, and we can filter correlated variants by testing for heterogeneity of genetic effects in different population samples. This approach formalizes the descriptive comparison of p-values that has typified similar cross-population fine-mapping studies to date. We applied this method to correlated SNPs in the cholinergic nicotinic receptor gene cluster *CHRNA5-CHRNA3-CHRNB4*, in a case-control study of cocaine dependence composed of 504 European-American and 583 African-American samples. Of the 10 SNPs genotyped in the r^2 ^≥ 0.8 bin for *rs16969968*, three demonstrated significant cross-population heterogeneity and are filtered from priority follow-up; the remaining SNPs include *rs16969968 *(heterogeneity p = 0.75). Though the power to filter out rs16969968 is reduced due to the difference in allele frequency in the two groups, the results nevertheless focus attention on a smaller group of SNPs that includes the non-synonymous SNP rs16969968, which retains a similar effect size (odds ratio) across both population samples.

**Conclusion:**

Filtering out SNPs that demonstrate cross-population heterogeneity enriches for variants more likely to be important and causative. Our approach provides an important and effective tool to help interpret results from the many GWA studies now underway.

## Background

Large-scale genome-wide association (GWA) studies for mapping genes for complex traits have now become a reality. Recent GWA studies have succeeded in discovering robust, novel findings of SNPs associated with human diseases including diabetes and breast cancer [1-5]. Even psychiatric diseases, notoriously challenging despite many well-designed family-based studies, have begun to reveal some of their genetic underpinnings to large-scale association designs [6,7].

These exciting successes are expected to ultimately lead to improved understanding of underlying biological mechanisms; this knowledge can then translate to prevention and treatment efforts. However, once an initial disease association study has been completed, any SNP significantly associated with the phenotype in fact represents a finding for all genetic variants correlated with that original SNP through linkage disequilibrium (LD), as measured by r^2^. Even after a successful replication study, a set of correlated SNPs is expected to replicate. Therefore, multiple variants become candidates for functional and biological follow-up of that signal. There is a need for approaches that can help direct laboratory follow-up efforts to the most promising and potentially causal variants.

We propose an approach that can help refine association signals and distinguish among these correlated variants. The overall idea is to follow up initial GWA signals in a population sample having a different population history from the initial "discovery" sample. The first step is to obtain genotypes in both population samples at the discovered associated SNP and at correlated SNPs in its r^2 ^bin. The second step is to perform formal heterogeneity testing to highlight variants that evidence similar genetic effect in both populations. To aid interpretation, an additional step would be to evaluate the power to filter out a SNP by the heterogeneity test under the assumption of a range of SNP allele frequencies in the second population. We propose specific ways to accomplish these steps in the methods below. This overall approach, which we call cross-population contrast mapping, works on the hypothesis that important biological mechanisms underlying disease are shared in common across human populations, although differences in allele frequencies at risk loci can lead to differences in prevalence in the different populations. When a risk allele has similar biological mechanism and exerts a similar effect on phenotype across these different populations, the contrasting linkage disequilibrium patterns in these populations can then be leveraged to narrow down the source of the association and identify the functional variant.

This general concept of mapping in different populations was recently applied to refine the association between three variants in *TCF7L2 *and type 2 diabetes [8]; there, European samples and African samples were analyzed separately and the results (p-values) were compared. Our contribution here is to propose a more systematic approach for studying these correlated SNPs within a given r^2 ^bin of interest, using the combined samples in a logistic regression framework that allows formal testing for heterogeneity of genetic effects across population groups. We then apply this method to a case-control cocaine-dependence dataset of European-Americans and African-Americans. In this application, the association signal we targeted is in the region of the *CHRNA5-CHRNA3-CHRNB4 *gene cluster. This gene cluster was originally reported to be associated with nicotine dependence [6], and has now shown evidence of association with cocaine dependence in European-Americans [9].

## Methods

### Study design and sample

Recruitment for the Family Study of Cocaine Dependence (FSCD) targeted equal numbers of men and women, and equal numbers of European-American (EA) and African-American (AA) participants. Cocaine dependent subjects were recruited from inpatient and outpatient chemical dependency treatment centers in the St. Louis area. Eligibility requirements included meeting criteria for DSM-IV cocaine dependence, being 18 years of age or older, speaking fluent English, and having a full sibling within five years of age who was willing to participate in the family arm of the study. Control subjects were recruited through driver's license records maintained by the Missouri Family Registry, housed at Washington University School of Medicine for research purposes. Controls were matched to cocaine dependent subjects based on age, ethnicity, gender, and zip code. Exclusionary criteria for controls included dependence on alcohol or drugs, including nicotine. Controls were required to have at least used alcohol in their lifetime because substance-abstinent individuals are considered phenotypically unknown; i.e., they may have a high genetic liability for addiction, but the absence of any substance use would preclude their progression to dependence. Blood samples for DNA analysis were collected from each subject and submitted, together with electronic phenotypic and genetic data, to the National Institute on Drug Abuse (NIDA) Center for Genetic Studies. The study obtained informed consent from all participants and approval from the appropriate institutional review boards.

The genetic arm of the FSCD consists of unrelated cases and matched unrelated controls from the FSCD who were genotyped. The genetic sample is composed of 504 EA participants (260 cases with DSM-IV cocaine dependence and 244 controls) and 583 AA participants (344 cases and 239 controls).

### SNP selection and genotyping

SNPs in the *CHRNA5-CHRNA3-CHRNB4 *gene cluster on chromosome 15 were selected for study. We targeted this region because of recent findings of strong association between SNPs across this cluster and nicotine dependence. In particular, here we have focused on a non-synonymous coding SNP in *CHRNA5*, *rs16969968*, and its genetic correlates, that is, SNPs having high r^2 ^with it. This SNP has demonstrated strong association with nicotine dependence [6,10] and has recently been replicated in independent samples, either through analysis of the same SNP [11] or proxy SNPs having very high r^2 ^with it [12,13]. This SNP or its r^2 ^proxies have now also demonstrated association with lung cancer [13-15].

This SNP *rs16969968 *is a high priority variant for potential functional effect: the change in amino acid 398 from aspartic acid (encoded by the G allele) to asparagine (encoded by A, the minor allele) results in a valence change, as noted in [6]. We also have recent data that this *D398N *amino acid change directly results in a change in function for the receptor [11]. Most important for the present study, *rs16969968 *has now been shown to be associated with cocaine dependence in two independent European-American samples from the FSCD and from the Collaborative Study on the Genetics of Alcoholism [9]. Interestingly, the risk allele for nicotine dependence appears to be protective for cocaine dependence, suggesting that the involvement of nicotinic receptors (*nAChR*s) in addiction is complex. The *nAChR*s are well known to be involved in both excitatory and inhibitory neurons impacting dopamine transmission, and this dual involvement provides biological plausibility for a bidirectional effect of the same genetic variant. Our goal in this report is now to examine the SNPs correlated with *rs16969968 *across the European American and African American samples in the FSCD to narrow and define the association signal.

For this cross-population mapping study, the analysis focused on 10 genotyped SNPs: *rs16969968 *and its genotyped correlates, defined by the r^2 ^≥ 0.8 bin (that is, the set of SNPs satisfying r^2 ^≥ 0.8) for *rs16969968 *in the HapMap CEU (Centre d'Etudie Polymorphisme Humaine (CEPH), Utah residents with ancestry from northern and western Europe) sample. These SNPs were part of a larger set genotyped in the FSCD sample by the Center for Inherited Disease Research (CIDR) with a custom Illumina SNP array to cover multiple candidate genes. Details of the CIDR genotyping procedures are available at their website .

### Population structure analysis

An additional 380 unlinked SNPs were genotyped for STRUCTURE [16] analysis to allow tests for the absence of confounding population structure. Using a two cluster solution computed in duplicate, no significant association between estimated cluster membership probability (inferred ancestries) and case-control status was found after accounting for self-reported race/ethnicity. Furthermore, these probabilities are perfect predictors of self-reported race. The same lack of association holds for three, four and five cluster solutions. These results indicate that our logistic regression framework described below, which analyzes the full sample while accounting for race as a covariate, is appropriate.

### Linkage disequilibrium

Linkage disequilibrium (LD) between SNPs was calculated using Haploview [17] (for HapMap data) and also the verbose option of ldmax [18] (for EA and AA cases and controls). Both CEU and YRI (Yoruba in Ibadan, Nigeria) HapMap data were used, separately. Plots for LD in the case-control data were generated using a custom program (A. Hinrichs, personal communication).

### Genetic association and heterogeneity analyses

Our primary single SNP association analyses of case-control status use logistic regression models, implemented using SAS (Cary, NC). To analyze the combined samples from two different population groups, terms are included in the base model to denote population source (*s*) and to correct for any necessary covariates (e.g. gender), denoted by variables *x*_*i*_, *i *= 1, ... *n*. In general, the non-genetic base model is then: $\ln\left(\frac{P}{1-P}\right)=\alpha_{0}+\alpha_{1}x_{1}+...+\alpha_{n}x_{n}+\beta_{1}s$, where *P *is the probability of being a case and *s *is sample race/ethnicity. In our particular application to the cocaine dependence data, we included two covariates (*n *= *2*), gender (0 = male, 1 = female) and year of birth, and the two populations are EA (*s *= *0*) and AA (*s *= *1*) from self-report. Genotype status *G *at each marker is modeled log-additively (multiplicatively) and coded as the number of copies of the minor allele in the European-American sample; this coding choice is arbitrary but allows for consistent reporting. The full model includes both genotype (*β*_2_*G) and genotype-by-population (*β*_3_*G*s) terms: $\ln\left(\frac{P}{1-P}\right)=\alpha_{0}+\alpha_{1}x_{1}+...+\alpha_{n}x_{n}+\beta_{1}s+\beta_{2}G+\beta_{3}Gs$ and we test for significance of genetic effect by the standard likelihood ratio chi-square statistic with two degrees of freedom (df). The population-specific odds ratios for the effect of a copy of the minor allele are given by exp(*β*_2_) for the EA sample and exp(*β*_2 _+ *β*_3_) for the AA sample.

If the overall test for significant genetic effect is significant, we then test for heterogeneity of genetic effect across the two population samples by comparing the above full model to the model $\ln\left(\frac{P}{1-P}\right)=\alpha_{0}+\alpha_{1}x_{1}+...+\alpha_{n}x_{n}+\beta_{1}s+\beta_{2}G$; that is, the interaction term is removed. If the resulting likelihood ratio chi-square statistic is significant, we conclude that the genetic effect is not similar in the two population samples and thus the SNP is lower priority for follow-up; the population-specific odds ratios defined above can then indicate how the effects differ in the two populations.

### Power Analysis

We are interested in the power to filter a SNP from consideration when the association is detected in one population but has no effect in the second population. This power depends on the allele frequency of the SNP in the second population and can be estimated by simulating the second population under an odds ratio of 1 and specified allele frequencies for the SNP, assuming Hardy-Weinberg equilibrium. It is important to note that no adjustment for LD is needed here; we are evaluating our ability to rule out the specific, genotyped SNP. The simulated data is then analyzed together with the real data at the SNP of interest for the first population, using the logistic regression approach above, and power to detect cross-population heterogeneity is estimated by the proportion of replicates which achieve a given significance level.

We derived the needed probabilities for this simulation as follows. Let *N*_1 _be the number of cases and *N*_2 _be the number of controls in the second population, and let *p *be the frequency of the A_1 _allele and *q *= *1-p *be the frequency of A_2_. Then we note that for the 2 × 3 table of case status by genotype status, the marginals for the case and control counts are *N*_1 _and *N*_2 _respectively, and the marginals for the A_1_A_1_, A_1_A_2_, and A_2_A_2 _counts are *p*^2^*(N*_1_+*N*_2_*)*, *2pq(N*_1_+*N*_2_*)*, and *q*^2^*(N*_1_+*N*_2_*) *respectively. Then the probabilities for the cells of the 2 × 3 table, recalling that the odds ratio is set to be 1 (corresponding to no effect in the second population), are the products of the marginal probabilities, so that $\mathrm{Pr}({\text{case and A}}_{1}{\text{A}}_{1})=\frac{N_{1}}{N_{1}+N_{2}}p^{2}$, $\mathrm{Pr}(\text{ctrl and }A_{1}A_{1})=\frac{N_{2}}{N_{1}+N_{2}}p^{2}$, etc.

For our example study, we therefore sampled from the above probabilities to simulate samples of 344 cases and 239 controls assuming minor allele frequencies of 0.3 down to 0.05, corresponding to the two extremes for power: where the allele frequency in the second population is similar to the EA frequency, and where it is notably lower as we see at rs16969968. Each simulated AA dataset was then combined with the real EA data at rs16969968. For simplicity, we used only the data at rs16969968, because this will estimate power to detect cross-population heterogeneity not only at rs16969968 itself, but also at the other SNPs because they have very high r^2 ^with rs16969968 in the EA sample. For each allele frequency, we generated 1000 replicates and recorded the proportion of replicates for which the cross-population heterogeneity was detected (i.e. the population*genotype term was significant) at fixed significance levels.

## Results

### Linkage disequilibrium

**Figure 1:**
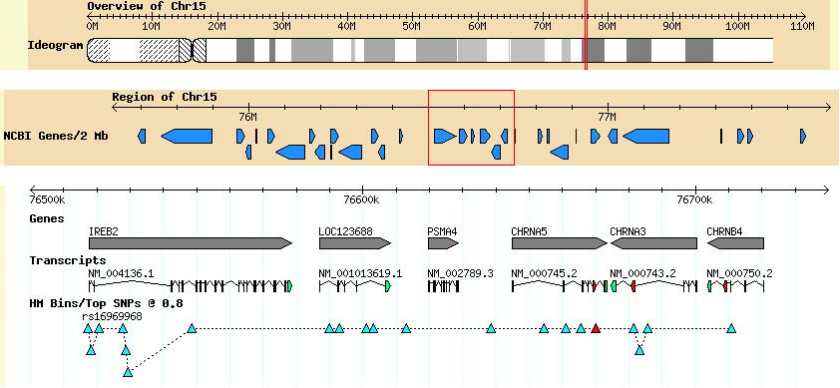
The r^2 ^≥ 0.8 bin for *rs16969968 *in HapMap CEU. The red triangle denotes *rs16969968*, and is connected to all other SNPs (triangles) in the r^2 ^bin by the dotted line. The vertical position of the triangles has no meaning, but allows all the SNPs to be shown without overlapping the triangles. The rs numbers for all SNPs in this bin are given in map order in Table 1.

**Figure 2:**
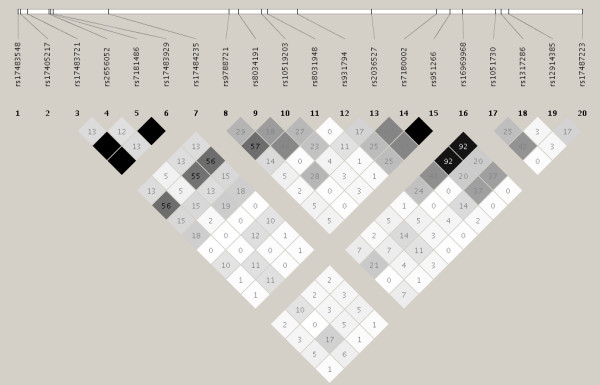
LD (r^2^) in the HapMap YRI sample, for the r^2 ^≥ 0.8 bin for *rs16969968 *defined by HapMap CEU LD. Of the 21 bin members pictured in Figure 1, 20 were genotyped in HapMap YRI of which 4 were monomorphic. The numerals in the cells denote the r^2 ^between the two SNPs corresponding to the cell. Cells with no numeral indicate r^2 ^= 1. Cell shading indicates strength of r^2 ^as shown by the numeral.

Figure 1 shows the *CHRNA5-CHRNA3-CHRNB4 *gene cluster at the top. The red triangle denotes the SNP *rs16969968*; the other triangles indicate all SNPs in HapMap correlated with it (r^2 ^≥ 0.8) in the CEU population sample. This figure thus indicates the "r^2 ^bin" of 21 SNPs that, according to HapMap CEU LD patterns, are not likely to be distinguishable even after a replication study in a similar population of European descent. Figure 2 shows the HapMap r^2 ^values among these same SNPs in the Yoruba (YRI) HapMap sample, and indicates that SNP correlations are indeed reduced in this sample. In YRI, only two small non-trivial r^2 ^≥ 0.8 bins remain, one composed of *rs17483721*, *rs7181486*, and *rs17483929*, and the other of *rs7180002*, *rs951266*, and *rs1051730*. The remaining polymorphic SNPs are singleton bins. One SNP, *rs17486278*, was not genotyped in the HapMap YRI sample, and four others were monomorphic in YRI: *rs17483548*, *rs17405217*, *rs17484235*, and *rs16969968 *(the *D398N *variant). The pairwise r^2 ^for the remaining 16 SNPs is displayed.

**Figure 3:**
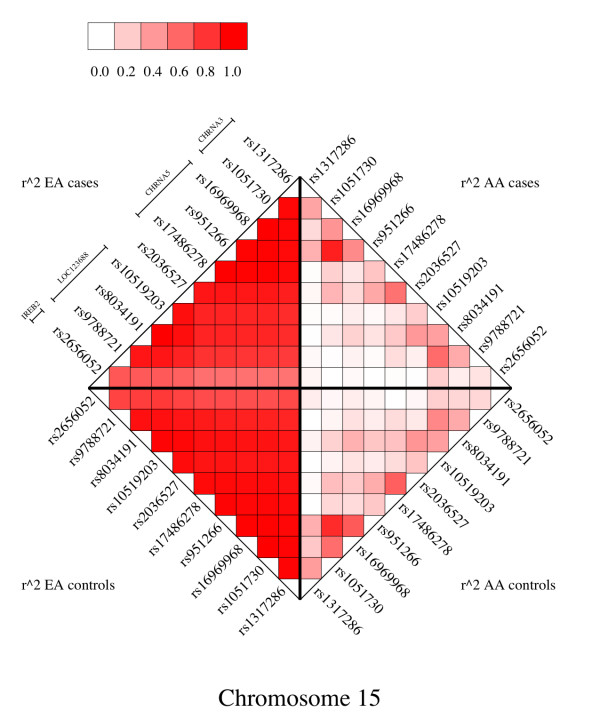
LD plot (r^2^) in our cocaine-dependence case-control samples. The quadrants display AA cases (upper right), EA cases (upper left) EA controls (lower left), AA controls (lower right).

Figure 3 displays r^2 ^values in our case-control sample in European-Americans and African-Americans, respectively. The SNPs are all those genotyped in our sample that have r^2 ^≥ 0.8 with *rs16969968 *in HapMap CEU. Figure 3 defines the set of SNPs for which we will carry out our cross-population approach, and indicates that there are indeed contrasts in the LD patterns in our EA and AA samples to enable this approach.

### Genetic association and heterogeneity analyses

Ten of the 21 SNPs in the HapMap CEU-determined r^2 ^≥ 0.8 bin for *rs16969968 *were genotyped in our sample. In addition, we examined additional SNPs across chromosome 15 that were genotyped in our sample and found no additional SNPs having r^2 ^≥ 0.8 with *rs16969968 *in our own data. Table 1 shows HapMap CEU and YRI allele frequencies for all 21 SNPs in the bin, and also EA and AA allele frequencies in our sample for the 10 genotyped SNPs.

**Table 1:** SNPs in the r^2 ^≥ 0.8 bin for rs16969968, sorted by genomic position, and their allele frequencies.

SNP	pos_bp	gene	SNP function	Minor allele frequency, HapMap CEU	Minor allele frequency, HapMap YRI	Minor allele, EA sample/ AA sample^1^	Minor allele frequency, EA sample	Minor allele frequency, AA sample
rs17483548	76517368	IREB2	LOCUS	0.408	0.00			
rs17405217	76518204	IREB2	INTRON	0.408	0.00			
rs17483721	76520786	IREB2	INTRON	0.408	0.175			
rs2656052	76527987	IREB2	INTRON	0.408	0.475	C/C	0.3313	0.4580
rs7181486	76528673	IREB2	INTRON	0.408	0.175			
rs17483929	76529431	IREB2	INTRON	0.408	0.175			
rs17484235	76548469	IREB2	INTRON	0.408	0.00			
rs9788721	76589924	LOC123688	INTRON	0.433	0.371	C/C	0.3452	0.3602
rs8034191	76593078	LOC123688	INTRON	0.433	0.142	C/C	0.3333	0.1595
rs10519203	76601101	LOC123688	INTRON	0.433	0.300	C/C	0.3353	0.3225
rs8031948	76603112	LOC123688	INTRON	0.432	0.103			
rs931794	76613235			0.433	0.292			
rs2036527	76638670			0.425	0.217	A/A	0.3323	0.2187
rs17486278	76654537	CHRNA5	INTRON	0.409	Not available	G/G	0.3294	0.2942
rs7180002	76661048	CHRNA5	INTRON	0.417	0.117			
rs951266	76665596	CHRNA5	INTRON	0.417	0.117	A/A	0.3274	0.0858
rs16969968	76669980	CHRNA5	NONSYNON	0.424	0.00	A/A	0.3274	0.0497
rs1051730	76681394	CHRNA3	SYNON	0.4	0.110	T/T	0.3270	0.0943
rs1317286	76683184	CHRNA3	INTRON	0.405	0.297	G/G	0.3323	0.2204
rs12914385	76685778	CHRNA3	INTRON	0.43	0.198			
rs17487223	76711042	CHRNB4	INTRON	0.433	0.067			

^1^Minor allele in EA sample is the same as the minor allele in the AA sample for all genotyped SNPs

Table 2 displays the primary association test results and the population heterogeneity results for the combined EA and AA samples (columns 9–12). For comparison, Table 2 also provides the association results when the EA and AA samples are analyzed separately using logistic regression with covariates gender and year of birth and a log-additive genetic model (columns 5–8).

**Table 2:** Cross-population association results.

	EA SAMPLE ONLY		AA SAMPLE ONLY		PRIMARY ANALYSIS OF THE COMBINED SAMPLE			
SNP	p-value^1^	Odds ratio (95% confidence interval)	p-value^1^	Odds ratio (95% confidence interval)	Primary^2^ p-value	EA Population -specific odds ratio^3^	AA Population -specific odds ratio^3^	P-value, population-by -genotype term (heterogeneity)

rs17483548								
rs17405217								
rs17483721								
rs2656052	0.0147	0.71 (0.539, 0.935)	0.9737	1.004 (0.793, 1.272)	0.04656	0.7074	1.0008	0.06031
rs7181486								
rs17483929								
rs17484235								
rs9788721	0.0031	0.662 (0.505, 0.870)	0.4084	1.108 (0.868, 1.415)	0.008229	0.6619	1.1045	0.005783
rs8034191	0.0052	0.679 (0.518, 0.891)	0.7846	1.046 (0.756, 1.449)	0.01903	0.6797	1.0389	0.04839
rs10519203	0.0035	0.667 (0.509, 0.875)	0.8629	1.022 (0.795, 1.315)	0.01312	0.6679	1.0208	0.02396
rs8031948								
rs931794								
rs2036527	0.0062	0.683 (0.520, 0.897)	0.5081	0.906 (0.677, 1.213)	0.01797	0.6837	0.9048	0.1676
rs17486278	0.0032	0.663 (0.504, 0.871)	0.3669	0.884 (0.676, 1.155)	0.007876	0.6616	0.8848	0.1356
rs7180002								
rs951266	0.0051	0.677 (0.515, 0.890)	0.8252	0.952 (0.617, 1.469)	0.01777	0.6761	0.9512	0.1894
rs16969968	0.0033	0.664 (0.505, 0.873)	0.2397	0.729 (0.430, 1.235)	0.006299	0.6636	0.7313	0.7479
rs1051730	0.0036	0.666 (0.507, 0.876)	0.6663	0.913 (0.604, 1.380)	0.01135	0.6636	0.9149	0.2016
rs1317286	0.0044	0.675 (0.515, 0.884)	0.9011	0.982 (0.736, 1.311)	0.01586	0.6745	0.9830	0.06096
rs12914385								
rs17487223								

The SNPs correlated with *rs16969968 *on chromosome 15 (r^2 ^≥ 0.8 in HapMap CEU) are sorted by genomic position. The heterogeneity test results are given in the rightmost column.

^1^One-degree of freedom test as described in methods.

^2 ^Two-degree of freedom test as described in methods.

^3 ^Population-specific odds ratio for the effect of one copy of the minor allele, from the combined analysis as defined in the methods.

We see that among the genotyped SNPs in Table 2, all are associated with case status with a primary p-value less than 0.05; this is expected since these SNPs are strongly correlated with *rs16969968*, which we know is associated with cocaine dependence in the EA sample. The key column in Table 2 is the last one, which gives the p-value for the test of the population-by-genotype interaction term and provides a test for heterogeneity of effect in the two population samples. We see that three SNPs (*rs9788721*, *rs8034191*, *rs1051948*) show significantly different effects in the two samples, and two others have p-values of 0.06 (rs2656052, rs1317286). The population-specific odds ratio in the AA group for each of these SNPs is essentially 1. Therefore our method would rule out these SNPs as likely causative variants and assign them lower priority. Of the remaining 5 SNPs, we observe heterogeneity p-values ≤ 0.2 for all except *rs16969968*, which has a heterogeneity p-value of 0.75 and odds ratios in the EA and AA groups of 0.66 and 0.73 respectively.

Our conclusion is that among these genotyped SNPs, *rs16969968 *should be prioritized for follow-up because it exhibits the strongest evidence for a comparable odds ratio in both the EA and AA groups; the other 6 SNPs that survived filtering also remain as potential SNPs of interest, while three have been ruled out. This similar odds ratio at rs16969968 is observed despite the fact that the A allele has very different allele frequencies in EAs and AAs (0.33 versus 0.05 respectively). In contrast, the flanking SNP *rs951266 *has a similar level of allele frequency discrepancy (0.33 versus 0.08, which leads to a reduction in r^2 ^between it and *rs16969968 *in the AA sample (Figure 3)), but has an odds ratio of 0.95 in the AAs versus 0.68 in the EAs. Thus a discrepancy in allele frequency on its own does not indicate whether the odds ratio for the allele effect will be similar or not in the two groups. By including a "population" covariate in the overall model, we can adjust for differences in population rates, and then test for the significance of the population-by-genotype interaction.

Note that none of the SNPs are significant when analyzed in the AA sample alone. However, this result underscores the advantage of using the population-by-genotype term in the full sample. A non-significant result for a SNP in the AA sample does not allow us to rule it out; it is only when we test for significant cross-population heterogeneity in the full sample that we come to the useful conclusion that three of the ten SNPs in fact can be filtered from further priority consideration.

The above advantage is also clear when we consider that power in the separate AA sample is impacted by allele frequency differences. For a "true" locus having the allele frequency pattern of rs16969968, a large AA sample would be needed to ensure a significant result in EAs and also in AAs separately, because of the low MAF in AAs. For example, using standard power calculations for an allelic test [19], assuming the observed allele frequencies of 33% in EAs and 5% in AAs, prevalence of 3%, and a genotypic odds ratio of 1.4, our sample sizes attain 75% power at an alpha of 0.05 in the EAs, compared to only 27% power in the AAs. Nevertheless, our available sample sizes still allow useful filtering of the correlated set.

There are additional SNPs correlated with *rs16969968 *according to the current HapMap CEU build that were not genotyped and analyzed in our samples; one of these additional SNPs (*rs7180002*) was in fact attempted by CIDR but removed during quality control due to poor clustering (more than 3 genotype clusters). However, it is useful to note that according to HapMap YRI, *rs7180002 *is in perfect LD (r^2 ^= 1) with the genotyped *rs951266 *(Figure 2) and therefore is very likely to have association and heterogeneity results very similar to those of *rs951266*. For the other non-genotyped SNPs, the YRI LD patterns do not indicate strong correlations with our genotyped SNPs, and we therefore cannot draw conclusions for these.

### Power analyses

Table 3 shows the power to filter out a SNP when its allele frequency in the second population ranges from 0.3 down to 0.05, assuming that there is no genetic effect (odds ratio of 1) in the second population. As expected, power is reduced as the minor allele frequency decreases: when the allele frequency p = 0.3, power to detect a significant population-by-genotype interaction at the α = 0.05 level is 61% compared to power of 25% when p = 0.05. Recruiting a larger sample of African Americans would allow us to further refine the region of association and make a more definitive conclusion about rs16969968. Nevertheless, with only approximately 61% power even when p = 0.3, in our data we have good evidence to be able to rule out SNPs such as rs9788721 and rs10519203 (Table 2). Note furthermore that rs8034191 is filtered out (heterogeneity p-value of 0.048) despite having a MAF of only 0.15 in the AA sample, corresponding to an estimated power between 34% and 52% at the α = 0.05 level.

**Table 3:** Power results^1^

Minor allele frequency in 2^nd ^population	Significance level (α) required	Power (%) to detect significant population*genotype interaction^2^
0.3	0.1	73.9
	0.05	60.8
0.2	0.1	67.9
	0.05	51.7
0.1	0.1	49.6
	0.05	34.2
0.05	0.1	34.1
	0.05	24.6

^1^From simulation, assuming rs16969968 has no effect and a range of allele frequencies in the second population sample of 344 cases and 239 controls.

^2^When the population*genotype interaction term is significant, we filter the SNP from priority consideration.

## Conclusion and discussion 

After a large-scale association study, the set of SNPs associated with disease will typically include correlated SNPs. There is a need for approaches that can help determine if a particular SNP among a correlated set is likely to be the biologically causative SNP and thus focus follow-up efforts in the laboratory on these most promising variants. One approach is to consider these correlated SNPs and systematically prioritize them according to known genomic and biological information such as locations of genes and cross-species conserved regions [20]. Here we have implemented a complementary method to prioritize SNPs that capitalizes on differences in LD patterns between different world population groups. We first identify the SNPs in strong LD with an associated SNP as measured by the correlation coefficient r^2 ^in the initial population; when r^2 ^is reduced in a second population, we then have the opportunity to refine the association and filter out some of the originally correlated variants.

This method presumes that the mechanism of action for functional genetic variants is shared in common across human populations, although differences in allele frequencies at risk loci can lead to differences in prevalence. This premise is supported by an empirical study by Ioannidis et al. that showed that while the frequency of genetic markers may vary across populations (58% showed large heterogeneity), their biological impact on the risk for common diseases appears to be usually consistent across different 'races' (only 14% showed large heterogeneity in the genetic effects) [21]. Our analysis, which has demonstrated successful filtering of some of the SNPs correlated in the EA population, raises the interesting possibility that among these 14% of markers observed by Ioannidis to have heterogeneity of genetic effect, some fraction may still indeed correspond to gene variants that do have similar biological mechanism and genetic effect, but were represented by a SNP merely in LD with the true causal variant. Thus the proportion of common diseases for which the biological effects of the causative genetic variants are consistent across traditional racial groupings may perhaps be even higher than estimated in [21].

By design, our method chooses to focus attention on these shared biological mechanisms, rather than on possible population-specific mutations which may exist in some situations. This statistical approach filters association signals and allows likely functional alleles to be better defined. Thus the next steps of functional follow-up can be focused on a smaller, enriched pool of potential causative variants.

In this context, the correlation coefficient r^2 ^is the appropriate measure of LD rather than D'. The goal is to distinguish among SNPs that are statistically correlated, so that the disease association signal at each bin member in fact can be *statistically *explained by the LD between the SNPs, yet the *biological *basis of the observed association may potentially be due to any member of the bin. Our approach therefore allows us to filter out bin members to narrow down to the most likely biologically causative variants.

Our approach uses logistic regression, which is a classical tool for genetic association studies. We chose the 2-df test as our primary association test simply to allow for potential differences in populations up front. The key point is that this framework then allows for formal testing of heterogeneity that can be used specifically to filter across the correlated, associated variants. Logistic regression can test for heterogeneity of SNPs regardless of their correlations with each other, and has been used in the literature to analyze uncorrelated SNPs, in distinct genes, to confirm agreement of association results across datasets. However, the extension of analysis to highly correlated SNPs in the r^2 ^bin, as well as to populations having differing LD structure, not only allows filtering of correlated, significant signals (an important goal), but also can help prevent "false negative" findings of apparent non-consistency when a locus does indeed have consistent biological effect across populations. For example, suppose a study in one population detects association at a single genotyped SNP but ignores correlates, so that only the same SNP is tested using logistic regression in a second population. This SNP may not appear to be consistent because of LD differences, whereas a correlated, causative SNP might have shown clear association in both populations, had it been genotyped and tested.

The effectiveness of this method of course depends on the available genotyping coverage of the target region. If the "true" disease susceptibility locus is not genotyped, for example if it lies on a haplotype defined by genotyped SNPs, it will not be recognized and included among the enriched set of SNPs after cross-population filtering. However, the method still can narrow down the possibilities by eliminating individual SNPs. Furthermore, as genotyping and sequencing costs continue to decline, we expect that studies will be able to thoroughly and directly assay the genetic variation and therefore apply this approach most effectively.

It is important to note that it is possible for a significant difference in odds ratio to occur not because the effect is lacking in the second population, but because it is significantly stronger. Therefore, significant results from the cross-population heterogeneity tests should be reviewed to check for such cases, and such SNPs for which there is a clear genetic effect in both populations should not be filtered from further study. For the 10 SNPs studied in our sample, this situation does not occur: the point estimate for the population-specific odds ratio in the AA sample is essentially one for all SNPs except rs16969968, as we had hoped to demonstrate for at least some SNPs. The direction of effect for rs16969968 in the AA sample also matches the direction in the EA sample (allele A is "protective").

Homogeneity across populations can also be evaluated using a stratified Cochran-Mantel-Haenszel analysis and testing for homogeneity of the odds ratio with the Breslow-Day test. In general, we expect the results to be similar to those obtained by our logistic regression analysis. In our dataset, the Breslow-Day test appeared slightly less sensitive and filtered out only two SNPs at an α of 0.05; the Breslow-Day p-value was 0.0092 for rs9788721, 0.0596 for rs8034191, and 0.0305 for rs10519203. We favor using the heterogeneity test within the logistic regression framework because it is a natural extension of the logistic regression analysis, without the population-by-genotype term, that is already popular for the analysis of GWAS data and has been used by our group [6,7] and others.

This cross-population contrast method, applied to European-American and African-American case-control samples, was successful in refining an association signal between SNPs in the *CHRNA5-CHRNA3-CHRNB4 *gene cluster and cocaine dependence, with the results highlighting a group of SNPs that includes *rs16969968*, a non-synonymous coding SNP in *CHRNA5*. Among the 10 genotyped SNPs, which are highly correlated in populations of European descent, three are filtered out due to significant evidence for heterogeneity of association in the two population samples, two others have p-values of 0.06, and the remaining SNPs include rs16969968, which had heterogeneity p-value 0.75 and similar odds ratios in EAs and AAs. This result further highlights this variant as a potential causative variant, as it adds a new line of evidence – no significant heterogeneity between populations for *rs16969968 *– to existing knowledge that this SNP causes an amino acid change and is conserved across species. This interpretation must be tempered by the fact that the power to rule out this SNP is reduced due to the considerable allele frequency difference in the EA versus AA samples. With a larger sample size, better discrimination among the remaining SNPs might have been possible. Nevertheless, in the available data our approach has narrowed the field of candidate variants for functional follow-up. Indeed, our recent work has now shown that rs16969968 alters receptor function in vitro [11].

These results are especially interesting in light of recent publications that report association between variation in *CHRNA5-CHRNA3-CHRNB4 *and smoking quantity [12] and nicotine dependence [13] and thus provide evidence of replication of the initial association discovered in [6]. Furthermore, the same or correlated variants also show association with lung cancer [13-15]. In these new papers, the reported association was either at rs16969968 (when genotyped), or at rs8034191, rs1317286, or rs1051730, all of which have very high r^2 ^with rs16969968 (0.966, 1.0 and 0.90 respectively in HapMap CEU) and all of which we studied here. Our analysis studied cocaine rather than nicotine dependence, so the conclusions here may not directly translate to smoking or lung cancer. However it is intriguing to note that in our cocaine dependence analysis, rs8034191 is ruled out (heterogeneity p-value 0.048), rs1317286 has a heterogeneity p-value of 0.06, and rs1051730, while not ruled out (heterogeneity p-value 0.20), has an odds ratio of 0.91 in the AA sample versus 0.66 in the EA sample. Applying this cross-population approach to an African American sample of nicotine dependent (or lung cancer) cases and controls will be an important next step to further understand and dissect this strongly replicated association between the r^2 ^bin for rs16969968 and those diseases.

The *D398N *amino acid change at *rs16969968 *demonstrates large differences in allele frequency across populations. The minor allele frequency (MAF) is 33% in our EA sample and only 5% in our AA sample. In the HapMap Yoruba sample, *rs16969968 *is monomorphic, so our African American sample likely demonstrates population admixture. Though homogeneous samples have been promoted as a resource to increase power to detect association, it is with outbred and admixed samples and two different populations (European-American and African-American) that we have narrowed an association signal to a likely functional variant involved in substance dependence. These results underscore the importance of expanding current genetic disease mapping studies to include diverse population samples beyond those of European descent.

## Authors' contributions

NLS designed and carried out the statistical analyses and power calculations and drafted the manuscript. SFS carried out SNP selection for genotyping and contributed to the design of the study. AMG contributed to the conception and design of the study. RAG carried out the population structure analyses. ALH performed linkage disequilibrium analyses and visualization. JPR contributed to the conception and design of the study and advised on the implementation of the method. LJB contributed to the conception and design of the study and is the PI of the FSCD project that provided the samples. All authors contributed to the interpretation of results and the intellectual content of the manuscript, and have read and approved the final manuscript.
